# Therapeutic mechanisms of Lycii Fructus in male infertility: a comprehensive review

**DOI:** 10.3389/fphar.2025.1613156

**Published:** 2025-09-22

**Authors:** Dongyue Ma, Dexiu Li, Guanchao Du, Shengjing Liu, Anmin Wang, Hongyuan Chang, Hui Lv, Hao Wang, Fu Wang, Jun Guo

**Affiliations:** ^1^ Xiyuan Hospital of Clinical Medical College of Beijing University of Chinese Medicine, Beijing, China; ^2^ Department of Andrology, Xiyuan Hospital, China Academy of Chinese Medical Sciences, Beijing, China; ^3^ Department of Cardiology, Xiyuan Hospital, China Academy of Chinese Medical Sciences, Beijing, China

**Keywords:** Lycii Fructus, Lycium barbarum, male infertility, testis, sperm quality, oxidative stress, reproductive protection

## Abstract

**Background:**

Lycii Fructus (dried goji berry), the dried ripe fruit of *Lycium barbarum L*., has long been used in traditional Chinese medicine for its functions of tonifying the kidney, nourishing essence, soothing the liver, and improving vision. It has been widely applied to enhance male reproductive function. In recent years, modern pharmacological studies have revealed that Lycii Fructus is rich in various bioactive metabolites, particularly *Lycium barbarum* polysaccharides, betaine, carotenoids, and flavonoids, which exhibit antioxidant, anti-apoptotic, and hormone-regulating effects. This review aims to systematically summarize existing clinical and mechanistic studies on the protective effects of Lycii Fructus and its key metabolites on male infertility.

**Methods:**

A literature survey was conducted on studies reporting the effects of Lycii Fructus and its bioactive metabolites on male reproductive parameters, including spermatogenesis, hormone regulation, testicular structure, and molecular signaling pathways.

**Results:**

Evidence suggests that Lycii Fructus and its metabolites can improve male reproductive function and sperm quality by modulating the hypothalamic–pituitary–gonadal axis, alleviating oxidative stress, inhibiting testicular cell apoptosis, suppressing pro-inflammatory factors, reducing testicular fibrosis, and regulating autophagy. Animal studies have shown that these effects may be mediated through key signaling pathways such as PI3K/Akt, SIRT1/Nrf2, and AMPK/PGC-1α, thereby enhancing testicular steroidogenesis and antioxidant capacity, among other benefits, and mitigating reproductive damage induced by diabetes, obesity, radiation, and environmental toxins. Although clinical evidence supporting the use of Lycii Fructus alone to improve male infertility is still lacking, traditional Chinese medicine compound formulas containing Lycii Fructus have demonstrated good efficacy and safety in treating oligoasthenozoospermia. Given the increasing attention to the safety and preventive health potential of natural botanical medicines, Lycii Fructus is emerging as a promising natural therapeutic agent for the treatment of male infertility.

**Conclusion:**

Lycii Fructus and its metabolites show promising therapeutic potential for male infertility by improving sperm quality, protecting testicular structure and function, and mitigating damage induced by various stressors.

## 1 Introduction

Male reproductive health is a vital component of overall wellbeing, yet it is increasingly challenged by modern lifestyle factors and environmental toxins (Daniels and Berger Eberhardt, 2024). It is estimated that male factors contribute to approximately 50% of infertility cases, which not only severely affect quality of life but also impose substantial psychological and economic burdens on individuals and healthcare systems (Minhas et al., 2021). Although conventional therapies such as hormonal treatments and assisted reproductive technologies (ART) are available, their high costs and potential side effects have sparked growing interest in alternative and complementary treatment options (Liu et al., 2024b; Zhang et al., 2024). Chinese botanical medicine has a long-standing history in the treatment of male reproductive disorders, offering a variety of natural metabolites with potential therapeutic activity (Wang et al., 2020). Among these, *Lycii Fructus*—the dried fruit of *Lycium barbarum L.* or *Lycium chinense Mill.* from the Solanaceae family—is widely used in traditional Chinese medicine (TCM) for its nourishing and restorative properties (Yu et al., 2023).

Lycii Fructus is officially recorded in the *Pharmacopoeia of the People’s Republic of China* (2020 Edition) as the dried ripe fruit of *Lycium barbarum* L. (Solanaceae). The fruits are harvested in summer and autumn when they turn red. Commonly known as “goji,” this medicinal plant has been documented as early as 200–250 AD, which describes its ability to “tonify the kidney, replenish essence, brighten the eyes, and strengthen the body” (Wenli et al., 2021). According to TCM theory, the kidney is considered the root of innate vitality, governing reproduction and development. Lycii Fructus are frequently used to tonify kidney essence and address deficiencies related to reproductive health (Hu et al., 2024). Modern pharmacological studies have isolated several key active metabolites from goji berries, including *Lycium barbarum* polysaccharides (LBP), zeaxanthin, betaine (BET), and flavonoids. These metabolites exhibit multiple bioactivities, such as antioxidant, anti-inflammatory, and hormone-regulatory effects (Yu et al., 2023). Recent research suggests that these bioactive substances may help preserve male reproductive health by improving sperm quality, increasing testosterone (T) levels, and combating oxidative stress—an important pathogenic mechanism implicated in infertility (Luo et al., 2011). With the growing demand for natural and preventive health interventions, goji berries are emerging as a promising candidate for integration into holistic medical strategies. This article aims to systematically explore the mechanisms by which goji berries support male reproductive health and to elucidate their potential therapeutic value in the treatment of male fertility disorders.

## 2 Retrieval methods

### 2.1 Search strategy

With reference to previous systematic reviews on male infertility (Li et al., 2022; You et al., 2020), we developed relevant search terms (Table 1). We searched the PubMed, Web of Science, and other databases for relevant studies up to April 2025. Additionally, we reviewed the reference lists of the articles identified through our search strategy and selected relevant literature based on their keywords.

**TABLE 1:** Electronic search strategies.

No.	Search item
#1	“Lycium barbarum” OR “Lycium barbarum polysaccharide” OR “Lycium barbarum extract” OR “Lycium” OR “Lycium chinense” OR “Wolfberry” OR “Wolfberries” OR “Goji” OR “Lycii fructus” OR “Gouqizi”
#2	“male reproduct*” OR “sperm” OR “semen” OR “testis” OR “testes” OR “testicular” OR “male infertility” OR “male fertility” OR “asthenozoospermia” OR “male subfertility” OR “male sterility” OR “oligospermia” OR “oligoasthenozoospermia” OR “asthenospermia” OR “azoospermia”
#3	#1AND #2

### 2.2 Eligibility criteria

#### 2.2.1 Inclusion criteria

We conducted a comprehensive and systematic search for studies following the guidelines outlined in the Preferred Reporting Items for Systematic Reviews (PRISMA) statement. The review process was structured using the PICO (Participants, Intervention, Comparison, and Outcomes) framework as detailed below:

Participants: Males with infertility (oligo/astheno/teratozoospermia, idiopathic) or animal models of impaired fertility.

Intervention: Studies involving patients or animals treated with Lycii Fructus or its extracts must clearly provide the Latin scientific name (*Lycium barbarum L*.) and collection origin of the botanical material to ensure the use of the target species and exclude potential adulteration with closely related species.

Comparison: Placebo/no treatment/standard care.

Outcomes: Semen parameters (count, motility, morphology), pregnancy rate, Hormones, oxidative stress markers, sperm DNA fragmentation, *etc.*

#### 2.2.2 Exclusion criteria

The following studies were excluded: (1) nonclinical studies or studies not involving animal experiments (e.g., review articles, case reports, letters, comments, posters, book chapters, *etc.*); (2) duplicate studies and studies with incomplete data; (3) studies not primarily focusing on Lycii Fructus; (4) studies not primarily focusing on male fertility disorders.

### 2.3 Data collection and analysis

#### 2.3.1 Selection of studies

Two authors (Dongyue Ma and Dexiu Li) searched for relevant articles according to the search terms and summarized the results. Original articles involving Lycii Fructus for male fertility disorders were included, and duplicate studies were eliminated. Some studies were excluded after analyzing the title, abstract, and full text. The reference lists of each study were also checked when necessary to include relevant research that might have been missed in the initial search. Dissenting opinions were submitted to another author (Guanchao Du) for adjudication throughout the entire process.

#### 2.3.2 Data extraction

**FIGURE 1:**
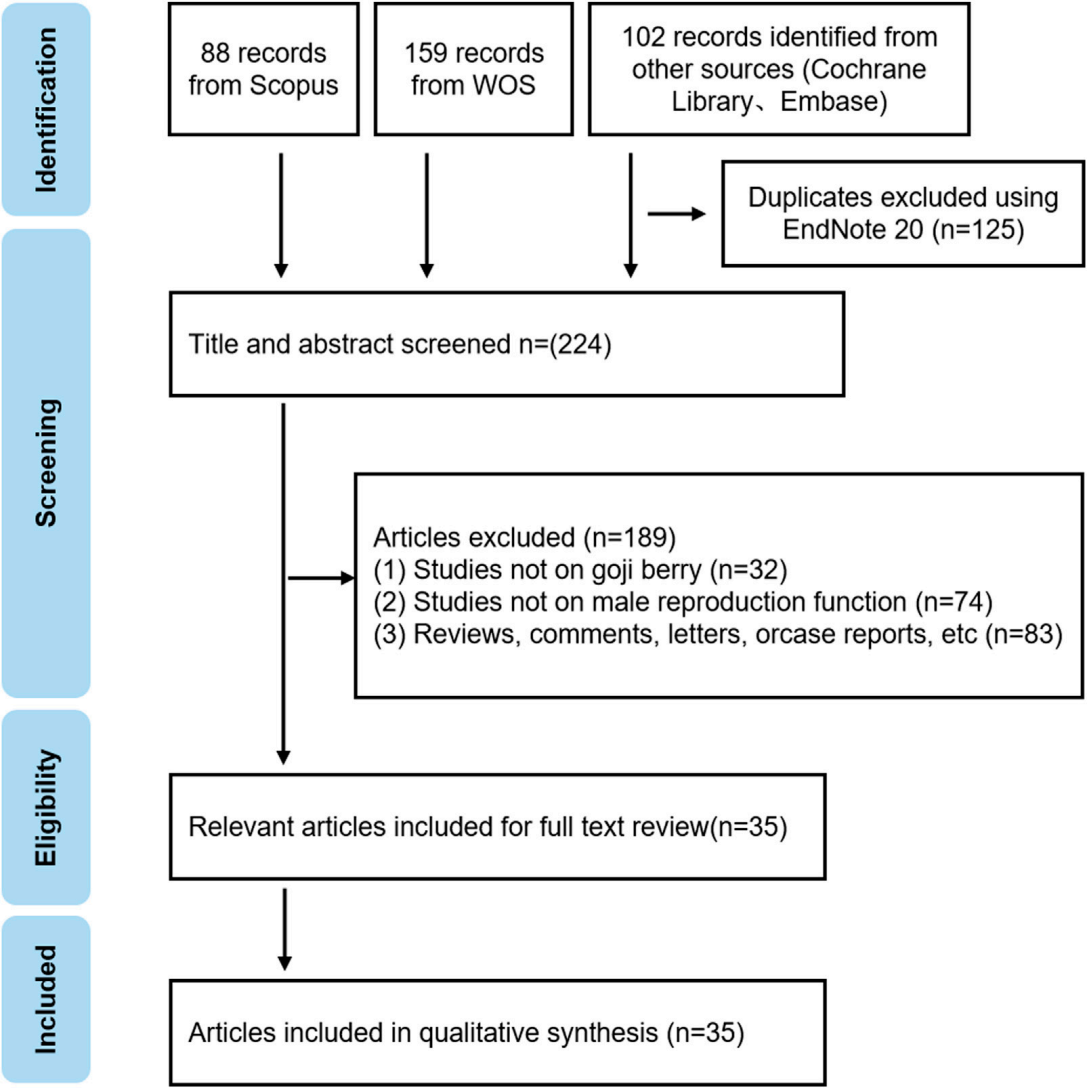
Flow chart of the retrieval.

Dongyue Ma and Dexiu Li independently extracted data in a standardized format, including study characteristics, interventions, and results. To ensure accuracy, cross-verification was performed, and any discrepancies were resolved through discussion with a third author (Guanchao Du) (Figure 1).

## 3 Chemical metabolites, pharmacology, potential applications, and toxicity of goji berries

**FIGURE 2:**
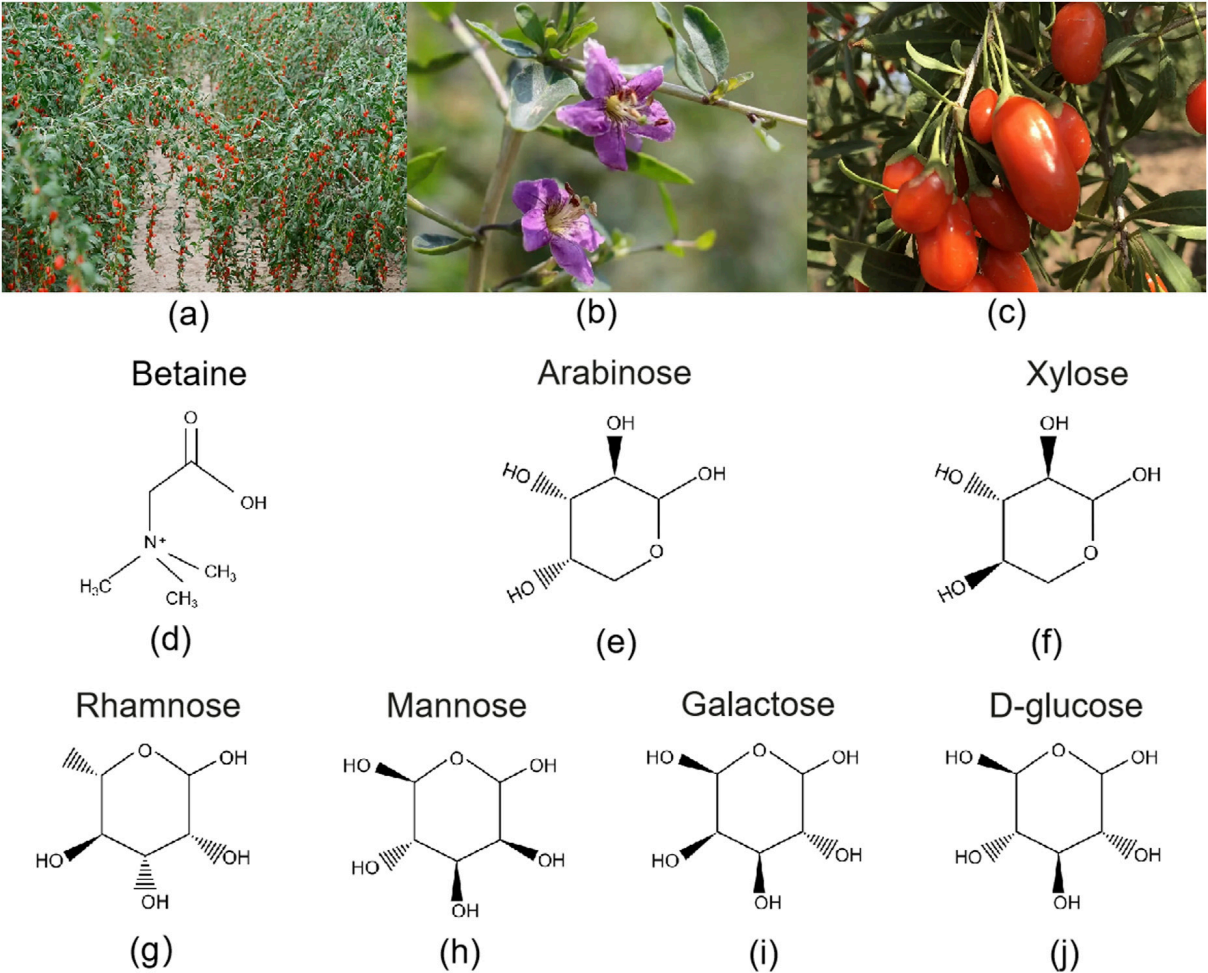
Botanical features and key bioactive compounds of Lycii Fructus. **(a)** Goji tree with mature fruits, showing its characteristic shrub-like growth habit and elongated red berries; **(b)** Close-up of a Goji flower, highlighting its delicate purple corolla and prominent stamens; **(c)** Fresh ripe goji berries, oblong in shape with smooth, waxy surface, 6–8 mm in diameter; **(d–j)** Chemical structures of representative metabolites found in Lycii Fructus. Betaine (Trimethylglycine, a quaternary ammonium alkaloid); Arabinose (Aldopentose, commonly in furanose form in natural polysaccharides); Xylose (Aldopentose, C-3 epimer of arabinose); Rhamnose (6-Deoxyhexose, L-configuration); Mannose (C-2 epimer of glucose, aldohexose); Galactose (C-4 epimer of glucose, aldohexose); D-glucose (Pyranose-form aldohexose, monomer unit of goji polysaccharides).

Lycii Fructus, derived from the dried mature fruit of *Lycium barbarum* L. (Figure 2). In TCM, it is recognized for its ability to nourish the liver and kidneys, replenish essence, and improve vision. It is widely used in wellness practices and as an adjunctive treatment for chronic diseases. Beyond China, Lycii Fructus are also used in traditional medicine or functional foods in countries such as Korea, Japan, parts of Eastern Europe, and North America (Teixeira et al., 2023).

Various parts of the goji plant—including the fruit, leaves, seeds, and root bark (Cortex Lycii)—have documented medicinal value. Traditionally, they have been used in decoctions, infusions, or formulated products to alleviate symptoms such as blurred vision, fatigue, and diabetes (Cui et al., 2020; Li et al., 2019). Over the past 2 decades, researchers have isolated and identified more than 200 chemical metabolites from goji berries, including LBP, BET, flavonoids, carotenoids (especially lutein and zeaxanthin), phenolic acids, organic acids, and amino acids (Jiang et al., 2024; Yu et al., 2023). Among these, LBP are the most representative metabolites (Figure 2). They possess complex structures with a wide molecular weight distribution and exhibit a broad range of biological activities, such as immunomodulation, antioxidant effects, anti-aging properties, hypoglycemic effects, and neuroprotection (Liu et al., 2022; Wang et al., 2024). Carotenoids represent the second major class of metabolites in goji berries, playing a protective role in the macular region of the eyes (Gao et al., 2017). Studies have shown that the antioxidant capacity of goji beverages depends not only on the content of polysaccharides and carotenoids but also on the synergistic effects of total phenolics and flavonoids (Mejri et al., 2023; Pires et al., 2018).

Moreover, modern processing technologies—such as enzymatic hydrolysis, microwave-assisted extraction, and ultrasonic extraction—have significantly enhanced the extraction efficiency and bioavailability of active metabolites (Liu et al., 2024a). Pharmacological studies of Lycii Fructus extracts have demonstrated multiple effects in animal and cell models, including immune regulation, lipid and glucose metabolism regulation, anti-tumor, anti-fatigue, neuroprotective, hepatoprotective, and reproductive function-enhancing properties (Tian et al., 2019; Xing et al., 2016). Notably, LBP exert broad-spectrum anti-inflammatory, antioxidant, and anti-apoptotic effects by modulating signaling pathways such as TLR4/NF-κB, PI3K/Akt, and Nrf2/ARE (Liu et al., 2021; Peng et al., 2022; Xie et al., 2024). For instance, in hyperlipidemia models, LBP were shown to reduce serum total cholesterol and triglyceride levels while improving hepatic steatosis (Yan et al., 2019).

Today, goji-derived products are widely used in health foods, beverages, cosmetics, and medicinal formulations, making goji an important resource in the development of functional foods and natural medicines (Zhang et al., 2022). In particular, it holds great promise in the fields of eye health, chronic disease management in the elderly, sub-health condition adjustment, and anti-aging interventions. A 2020 clinical study showed that long-term intake of Lycii Fructus extract could delay degenerative changes in the macular region, highlighting its value in visual health (Kan et al., 2020). While goji berries have a long history of use as both food and medicine and are generally considered safe, potential toxicity and adverse reactions should not be overlooked (Potterat, 2010). Some studies suggest that goji berry extracts may interact with anticoagulants such as warfarin, warranting caution in drug compatibility (Rivera et al., 2012). Although there are no conclusive reports of mutagenicity or genotoxicity in humans, caution is advised for pregnant or breastfeeding women, individuals with impaired liver or kidney function, and those considering long-term high-dose consumption.

**FIGURE 3:**
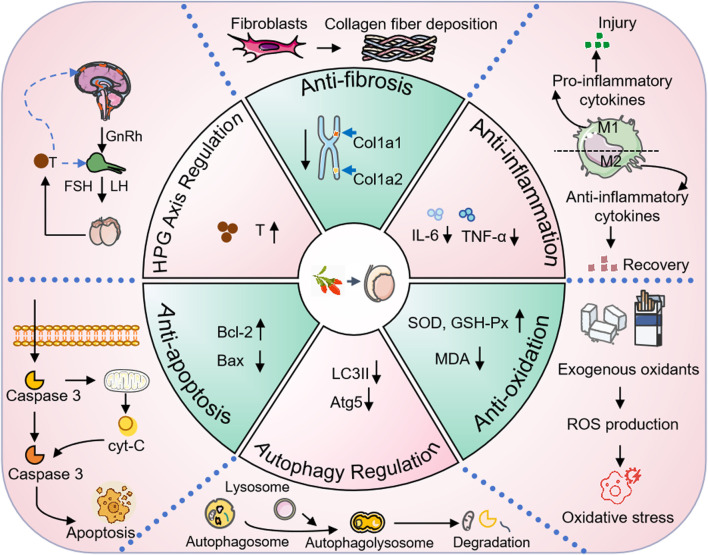
The protective effect of Lycii Fructus on male reproductive function. Abbreviation: cyt-c, Cytochrome c; IL-6, Interleukin-6; TNF-α, Tumor Necrosis Factor-alpha; SOD, Superoxide Dismutase; GSH-Px, Glutathione Peroxidase; MDA, Malondialdehyde; ROS, Reactive Oxygen Species; LC3II, Microtubule-Associated Protein 1A/1B-Light Chain 3-II; Atg5, Autophagy-Related Protein 5; HPG, Hypothalamic-Pituitary-Gonadal axis; GnRH, Gonadotropin-Releasing Hormone; FSH, Follicle-Stimulating Hormone; LH, Luteinizing Hormone; T, Testosterone; col1a1, Collagen Type I Alpha 1 Chain; col1a2, Collagen Type I Alpha 2 Chain.

In conclusion, as a botanical drug with both nutritional and medicinal properties, Lycii Fructus exhibit wide-ranging pharmacological activities and high application value (Figure 3). However, to ensure their safe, effective, and scientifically guided use, attention must be given to dosage control and population-specific suitability during further development and clinical application.

## 4 The relevant mechanism of action of Lycii Fructus in treating male infertility

In recent decades, accumulating pharmacological and experimental evidence has revealed that the beneficial effects of Lycii Fructus, especially its bioactive components such as LBP, extend far beyond simple nutritional supplementation. Instead, they encompass a range of biological activities targeting the key pathophysiological mechanisms underlying male infertility. These mechanisms include endocrine regulation via the hypothalamic-pituitary-gonadal (HPG) axis, antioxidant activity, anti-apoptotic and autophagy-modulating properties, inflammation resolution, and antifibrotic effects (Table 2). This section systematically reviews the current literature to elucidate the multi-targeted and multi-pathway actions of Lycii Fructus and its major metabolites in male reproductive protection and the restoration of fertility.

**TABLE 2:** Experiments on the effect of Lycii Fructus on male infertility.

Disease	Intervention	Mechanism	Action pathway or mediator site	Modeling method	Result	Category	Ref.
Fertility impairments	LBP (10, 20, 40 mg/kg/day) administered daily by intragastric gavage for 62 consecutive days	Regulating the HPG axis	↑Serum T, FSH and LH	A Type-1 diabetic model was induced in mice using 45 mg/kg streptozotocin (STZ) via intraperitoneal injection for 5 days	↑Johnsen’s testicular score and fertility levels	*In vivo*	Shi et al. (2017b)
Spermatogenesis dysfunction	Intraperitoneal injection of 200 mg/kg BET for 14 days	Anti-oxidation Anti-inflammation Anti-apoptosis	↑GSH ↓MDA and GSSG. ↓Apoptotic tubule and cell indices ↓IL-6 and TNF-α ↑AR, CD29 and INSL3	CRS induced in 8-week-old male C57BL/6J mice by fixation for 6 h a day for 35 days Anti-apoptosis	↑Johnsen’s score, seminiferous tubule diameter and spermatogenic cell arrangement ↑Sperm quality	*In vivo*	Meng et al. (2022)
LOH	Goji berry extract administered in 150 or 300 mg/kg/day	Anti-oxidation Anti-apoptosis	↑serum T and AR. ↓8-OHdG ↑SOD, Bcl-2/BAX, P-Akt and ERK	18-month-old male SD rats	↑Sperm motility	*In vivo*	Jeong et al. (2020)
Impaired reproductive system	Male mice received intragastric administration of LBP at doses of 0.2, 0.4, 0.6 g/kg	Anti-oxidation	↑Protein content and SOD activity ↓NO	Male Kunming mice were administered cyclophosphamide (28 mg/kg) daily for 5 days	↑Sperm density, movement, and normal morphology rate	*In vivo*	Qian and Yu (2016)
Spermatogenic impairment	Male mice received intragastric administration of LBP at doses of 10 mg/kg	Anti-apoptosis Anti-oxidation Anti-fibrosis	↑T-AOC, SOD, ATP6V0D2, COX6A2 and Acrosin ↓The ratio of apoptotic germ cells ↓Col1a1 and Col1a2 ↑TNP2 and RPL31	Chronic low-dose ethanol exposure in Immp2l^+/−^ mice	↑Testicular degeneration	*In vivo*	Liu et al. (2020)
None specified	Dietary supplementation with 1% Goji berry for 60 days	No significant changes in anti-oxidant and anti-inflammatory parameters	NA	Rabbits fed commercial feed	↑Semen quality and reproductive tract histology	*In vivo*	Brecchia et al. (2023)
Spermatogenic dysfunction	Oral administration of LBP at doses of 10, 20, 40 mg/kg for 62 consecutive days	Anti-oxidation Anti-apoptosis	**↑**Antioxidant enzyme activities **↓** Caspase-3 **↑**Bcl-2/Bax ratio	Diabetic male mice induced by STZ-induced type-1 diabetes	↑Sperm parameters, reproductive organ weight and histological appearance	*In vivo*	Shi et al. (2017a)
Testicular toxicity	Oral administration of LBPs in doses of 0, 10.0, 33.3, 100 mg/kg for 35 days	Anti-oxidation	↑SOD and GSH-PPX. ↓MDA. ↑Serum T	Male mice were orally administered cadmium chloride (CdCl2) at a dosage of 5.0 mg/kg body weight for 35 days	↓Testicular toxicity ↑Sperm parameters and testicular histology	*In vivo*	Zhang et al. (2017)
Male infertility	50 μg/mL LBP treatment for 48 h	Regulation of ER stress Anti-apoptosis Inhibits autophagy	↑ T in MLTC-1 cells ↓p-PERK, p-eIF2α and ATF4 ↓Caspase 3, 7 and 12 ↓LC3II and Atg5	DDP-induced apoptosis and autophagy in Leydig MLTC-1 cells	↑The viability of MLTC-1 cells	*In vitro*	Yang et al. (2018b)
Testis spermatogenic injury	LBP administered by gavage at doses of 50, 100, 200 mg/kg body weight for 7 days	Anti-oxidation Anti-apoptosis	↑Serum SOD and GSH-pox ↓Serum MDA ↑Bcl-2/Bax in testicular tissue	BPA was subcutaneously injected into mice at a dose of 20 mg/kg body weight for 7 consecutive days	↑The weights of testis and epididymis	*In vivo*	Zhang et al. (2013)
Heat stress-induced male infertility	LBP (25, 50, 100 mg/L, 24 h)	Anti-apoptosis	↑AR and p-Akt in Sertoli cells **↑**Occludin and ZO-1 ↑Ki67 in testis ↓CK-18 in testis	Sertoli cells from SD rats were heat-stressed (43 °C, 20 min)	↑The viability of Sertoli cells and BTB integrity	*In vitro*	Hu et al. (2021a)
Sperm damage	The sample was incubated at 37 °C for 20 min in 1 mL of sperm culture solution containing 1,000 μg/mL LBP	Anti-apoptosis Anti-oxidation Preservation of mitochondrial function	↑Bcl-2 ↓Bax, city-C, and caspase-3 ↓ROS	Sperm samples were cryopreserved using a glycerol-egg-yolk-citrate agent	↑Sperm motility ↓DNA fragmentation	*In vitro*	Yan et al. (2020)
Testicular dysfunction	LBP was administered at a dose of 100 mg/kg per day for 6 weeks	Pro-proliferation Anti-apoptosis	↑PCNA ↑SIRT1 and HIF-1α	Diabetic model induced by STZ in rats	↑Testicular function and insulin resistance ↓Blood glucose	*In vivo*	Lei et al. (2020)
Obesity-associated male infertility	Administration of LBP to high-fat diet-induced obese mice for 35 days	Anti-oxidation Alleviation of ER stress Hormonal regulation	↓MDA, p-eIF2a, GRP78, and CHOP ↑SOD and GSH ↑Serum T ↑Insulin sensitivity ↓FBG, TC, TG, LDL-C and HDL-C	High-fat diet-induced obese mice model	↓Glucose levels and insulin resistance ↑Pathological damage	*In vivo*	Yang et al. (2020)
Ischemic reperfusion injury associated with testis torsion	Goji berry extract (100 mg/kg, i.p., 7 days)	Anti-oxidation	↑TAC ↓TOS and OSI	Testicular torsion was induced in SD male rats by 5 h ischemia followed by 6 h reperfusion	↓Ischemic reperfusion injury	*In vivo*	Dursun et al. (2015)
Testicular dysfunction	Vivo: Intragastric administration of LBSO at a dose of 1,000 mg/kg/day for 4 weeks Vitro: LBSO (25, 50, 100, and 150 lg/mL)	Enhancement of mitochondrial function Anti-oxidation	↑SIRT3, AMPK and PGC-1α ↑HO-1, SOD-1, SOD-2 ↑INHB and T	Male SD rats received subcutaneous D-gal (125 mg/kg/day) for 8 weeks TM4 cells were exposed to 200 mmol/L D-gal for 48 h	↓Testis injury	*In vivo* and *vitro*	Yang et al. (2021)
Diabetes-induced BTB dysfunction	Oral administration of BET at doses of 200–800 mg/kg to diabetic mice for 8 weeks	Anti-oxidation	**↓**ROS and MDA **↑**SOD, CAT, and GSH ↑ZO-1, Occludin, Claudin-11, N-cadherin, Connexin-43 ↓p38 MAPK	Male mice received intraperitoneal STZ injections (45 mg/kg for 5 days)	↑Testicular morphology and BTB integrity	*In vivo*	Jiang et al. (2019)
Testicular injury	0–50 mg/L LBP treatment was administered for 14 days	Anti-oxidation Hormonal regulation	↑Cyp11b expression and SOD ↓Cyp19a expression and MDA	Juvenile zebrafish were exposed to nonylphenol	↓Testicular injury	*In vivo*	Tang et al. (2017)
Impaired reproductive system	Gavage LBP5%, 10%, 15% (w/v) solutions (0.5, 1.0, 1.5 g/kg) once a day for 5 consecutive days	Improving immune response	↑Serum IL-2, IL-12, and TNF-α	Mice were administered cyclophosphamide to induce reproductive impairment	↑Sperm density, motility, and normal morphology rate in testis	*In vivo*	Qian (2019)
Reproductive system damage	10 mg/kg LBP dissolved in saline by gavage (3 mL/kg/d) 6 h before irradiation	Anti-apoptosis	↑Bcl-2 ↓Bax and mitochondrial membrane depolarization	Male Long-Evans rats exposed to low-dose 60Co-γ irradiation	↑Mating function and testis organ coefficient	*In vivo*	Luo et al. (2014)
Testicular damage	Heat exposure model: Daily gastric gavage with 10, 50, 100, and 200 mg/kg LBP for 14 days DNA damage model: 50, 100, 200, 400 μg/mL LBP Hemicastration model: Daily gastric gavage with 10 mg/kg of LBP for 21 consecutive days	Anti-oxidation Hormonal regulation	↑SOD ↑FSH, LH, and T ↓MDA and DNA damage	Heat exposure-induced rat testicular damage model H_2_O_2_-Induced DNA Damage in mouse Testicular cells model Hemicastrated rat sexual behavior and reproductive function model	↑Testis and epididymis weights ↑Sperm quantity and quality ↑Sexual behavior	*In vitro* and *vivo*	Luo et al. (2006)
Testicular damage	Oral dose of 300 mg/kg of LBP once daily for a duration of 30 days	Anti-oxidation	↓GSH and TEAC ↑3NT	Adult male rats injected with CdCl2 (4 mg/kg, i.p., once)	↑Seminiferous tubule morphology	*In vivo*	Varoni et al. (2017)
Impaired reproductive system and testicular damage	LBP administered via daily gastric gavage at 10 mg/kg for 20 days	Anti-oxidation Hormonal regulation Anti-apoptosis	↑SOD and Serum T ↓MDA ↓DNA Damage in testicular cells	Wistar rats received subchronic ^60Co-γ Irradiation to the testes	↓Testicular damage ↑Reproductive parameters	*In vivo*	Luo et al. (2011)
Testicular toxicity	LBP administered orally at 200 mg/kg daily for 10 days	Anti-oxidation Hormonal regulation	↓ MDA ↑GSH-Px and plasma T	Doxorubicin-induced testicular toxicity in Sprague-Dawley rats	↑Testicular weight, sperm quality and histopathological damage	*In vivo*	Xin et al. (2012)
Testicular degeneration	LBP administered at 500 μg/mL in the culture medium	Anti-oxidation Anti-apoptosis	↓Lipid peroxidation ↓Cyt-c reduction by superoxide radicals ↓Pre-apoptotic and apoptotic cells	Testicular Tissue Block Culture	↑ Sperm Motility ↓Degenerated germ cells ↑Preservation of Tubular Structure	*In vitro*	Wang et al. (2002)
Diabetic testicular dysfunction	LBP administered orally at 40 mg/kg daily for 62 days	Inhibits autophagy Anti-oxidation	↑ Serum T ↑SOD, GSH-Px, and CAT ↓MDA, Beclin-1 and LC3 mRNA ↑p-PI3K and p-Akt	Male Institute of Cancer Research mice, aged 6–8 weeks, with type 1 diabetes induced by STZ	**↑**Sperm parameters and Histopathologic Damage	*In vivo*	Shi et al. (2018)
Renal and testicular injury	LbGp was intragastrically administered to mice 6 h, daily for 14 days	Autophagy regulation Anti-apoptosis Anti-fibrosis	↑Renal autophagy in mice ↓Testicular autophagy in mice ↓SIRT1, FoxO3a, p38 MAPK and LC3 expression ↓Collagen fiber deposition in kidney and testis tissues	Mice were intragastrically administered DEHP (1,500 mg/kg) to induce toxicity	↑Liver and Kidney Function ↓Glomerular enlargement, erythrocyte infiltration, and seminiferous tubule dilation ↑Level of testicular germ cells	*In vivo* and *vitro*	Zhou et al. (2022)
NA	LbGp, 100 mg/kg/day, administered via intragastric gavage for 12 weeks	Hormonal regulation	↑SF-1, Star, CYP11A1, HSD3B1, HSD17B3 ↑T pregnenolone and DHT ↓TGF-β Signaling Pathway	Healthy male C57BL/6J mice (6 weeks old) Primary Leydig cells	Proliferation in Leydig cells	*In vivo* and *vitro*	Liang et al. (2025)
DEHP-induced male reproductive damage	LbGp (100 mg/kg per day) via intragastric administration for 12 weeks	Anti-ferroptosis	↑GPX4,SLC7A11, FPN1 and GSH ↑ZO-1,Vimentin, E-cadherin, Occludin ↑FSHR and AR in Sertoli cells ↓MDA, Fe^2+^, TFR, FTH1, ACSL4, HO-1 and PTGS2	DEHP-exposed C57 mice TM4 Sertoli cells treated with MEHP ± LBP	↑Testicular histology, sperm morphology, and Sertoli cell function	*In vivo* and *vitro*	Yang et al. (2024)

Abbreviations: LBP, *lycium barbarum* polysaccharide; BET, betulinic acid; LBSO, *lycium barbarum* seed oil; STZ, streptozotocin; DDP, cisplatin; BPA, Bisphenol A; CdCl_2_, Cadmium chloride; D-gal, D-galactose; TEAC, trolox equivalent antioxidant capacity; ROS, reactive oxygen species; GSH, glutathione; GSSG, oxidized glutathione; GSH-Px, Glutathione peroxidase; SOD, superoxide dismutase; CAT, catalase; MDA, malondialdehyde; T-AOC, total antioxidant capacity; TAC, total antioxidant capacity; TOS, total oxidant status; OSI, oxidative stress index; 8-OHdG, 8-hydroxy-2′-deoxyguanosine; PCNA, proliferating cell nuclear antigen; BAX, BCL2 Associated X; Bcl-2, B-cell lymphoma-2; Caspase, Cysteine-aspartic proteases; Cyt-c, Cytochrome c; LC3, Microtubule-associated protein 1A/1B-light chain 3; ATF4, Activating transcription factor 4; PERK, Protein kinase RNA-like ER, kinase; eIF2α, Eukaryotic translation initiation factor 2α; CHOP, C/EBP, homologous protein; GRP78, Glucose-regulated protein 78; ZO-1, Zonula occludens-1; AR, androgen receptor; T, testosterone; FSH, Follicle-stimulating hormone; LH, luteinizing hormone; INSL3, Insulin-like 3; INHB, Inhibin B; HIF-1α, Hypoxia-inducible factor 1-alpha; SIRT1, Sirtuin 1; SIRT3, Sirtuin 3; AMPK, AMP-activated protein kinase; PGC-1α, Peroxisome proliferator-activated receptor gamma coactivator 1-alpha; HO-1, Heme oxygenase-1; GPX1, Glutathione peroxidase 1; Nrf2, Nuclear factor erythroid 2–related factor 2; Keap1, Kelch-like ECH-associated protein 1; ATP6V0D2, ATPase H+ Transporting V0 Subunit D2; COX6A2, Cytochrome c oxidase subunit 6A2; TNP2, Transition protein 2; RPL31, Ribosomal protein L31; Cyp11b, Cytochrome P450 family 11 subfamily B; Cyp19a, Cytochrome P450 family 19 subfamily A; BTB, Blood-testis barrier; MLTC-1, Mouse Leydig Tumor Cells-1; TM4, Sertoli Cell Line TM4; FBG, fasting blood glucose; TC, total cholesterol; TG, triglycerides; LDL-C, Low-density lipoprotein cholesterol; HDL-C, High-density lipoprotein cholesterol; IL, interleukin; TNF-α, Tumor necrosis factor-alpha; MAPK, Mitogen-activated protein kinase; 3NT, 3-Nitrotyrosine.

### 4.1 Regulation of the hypothalamic-pituitary-gonadal (HPG) axis

**FIGURE 4:**
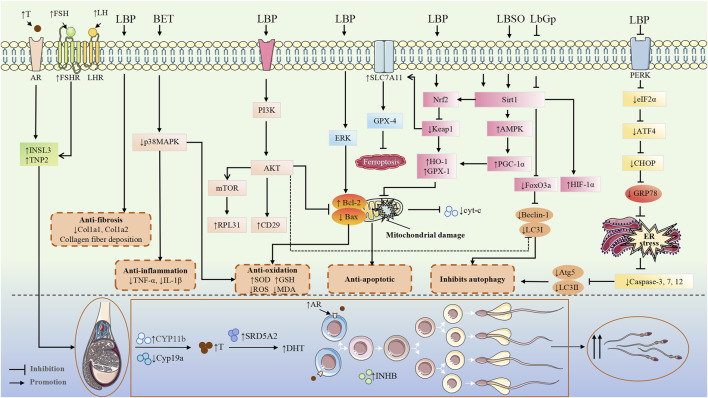
Mechanisms of the protective effects of Lycii Fructus in the treatment of male infertility. Abbreviations: LBP, *Lycium barbarum* polysaccharide; BET, betaine; LBSO, *Lycium barbarum* seed oil; LbGp, *Lycium barbarum* glycopeptide; INSL3, insulin-like peptide 3; cyt-c, cytochrome c; TNP2, transition protein 2; RPL31, ribosomal protein L31; CD29, cluster of differentiation 29 (integrin β1); Atg5, autophagy related 5; LC3II, microtubule-associated protein 1 light chain 3 II; CYP11b, cytochrome P450 family 11 subfamily B; Cyp19a, cytochrome P450 family 19 subfamily A (aromatase); GRP78, glucose-regulated protein 78; AR, androgen receptor.

HPG axis plays a central role in regulating male reproductive health by orchestrating the synthesis and release of key hormones, including gonadotropin-releasing hormone (GnRH) from the hypothalamus, luteinizing hormone (LH) and follicle-stimulating hormone (FSH) from the pituitary gland, and T from the testes. This tightly regulated axis governs the proliferation and maturation of germ cells, the maintenance of testicular architecture, and the overall capacity for spermatogenesis (Li et al., 2024). Disturbances in HPG axis function due to aging, chronic diseases, stressors, or exposure to endocrine-disrupting chemicals can impair spermatogenesis and T biosynthesis, leading to male reproductive dysfunction (Selvaraj et al., 2021). LBP, a major bioactive metabolite of Lycii Fructus, has been shown to exert protective effects against various etiologies of male reproductive dysfunction, primarily through modulation of the HPG axis. Experimental studies using 10, 20, and 40 mg/kg doses of LBP demonstrated its effectiveness in reversing diabetes-induced sexual dysfunction and fertility impairment in male mice, primarily through restoring hormonal balance (GnRH, LH, FSH, and T) (Shi et al., 2017b). However, the streptozotocin (STZ) -induced type 1 diabetes model used in this study, which causes complete β-cell destruction, may not accurately reflect human diabetes progression, potentially affecting the interpretation of LBP’s true efficacy (Furman, 2021). Additionally, the study’s failure to examine local testicular factors (e.g., oxidative stress and inflammatory markers) limits comprehensive understanding of LBP’s protective mechanisms and whether its reproductive benefits extend beyond HPG axis regulation. LBP administration (10 mg/kg) effectively restored heat exposure-induced reductions in T, LH and FSH levels while improving oxidative stress markers [increased superoxide dismutase (SOD) activity and decreased malondialdehyde (MDA) content] in the heat stress model. Similar treatment in hemicastrated rats significantly elevated serum T and lowered E2 levels without affecting LH/FSH, indicating potential direct effects on Leydig cells. Both models showed consistent dose-dependent responses with parallel improvements in sexual behavior and sperm quality (Luo et al., 2006). The study’s limitation in assessing key regulatory factors like GnRH, however, precludes definitive mechanistic conclusions. In another heat stress-induced testicular injury model, Hu et al. found that LBP could enhance the expression of Androgen receptor (AR) and Akt phosphorylation in Sertoli cells (Figure 4), stabilize the blood-testis barrier (BTB), and protect spermatogenic function, providing experimental evidence for the clinical prevention and treatment of male reproductive heat stress-induced injuries (Hu et al., 2021a).

In the context of aging-related hypogonadism, Lycii Fructus extract improves serum T levels and upregulates AR expression in testicular tissue, suggesting therapeutic potential in late-onset hypogonadism (LOH) without adversely affecting prostate volume or function (Jeong et al., 2020). In environmental toxicity models, such as juvenile zebrafish exposed to nonylphenol, the study found that LBP could ameliorate testicular damage by stimulating androgen secretion and enhancing antioxidant capacity (Tang et al., 2017). Moreover, LBP has been shown to restore T levels and mitigate androgen decline in animal models subjected to chronic ethanol exposure (Liu et al., 2020), restraint stress (Meng et al., 2022), and diet-induced obesity (Yang et al., 2020), further supporting its role in HPG axis homeostasis.

### 4.2 Antioxidant activity

Oxidative stress plays a pivotal role in testicular injury and male reproductive dysfunction. Excessive generation of reactive oxygen species (ROS) not only damages testicular tissue structure, impairs spermatogenic cells and the BTB, but also induces cell apoptosis and disrupts T synthesis, ultimately leading to decreased fertility (Leisegang et al., 2017). LBP has been extensively reported to exhibit potent antioxidant properties, thereby mitigating testicular injury and reproductive dysfunction caused by various environmental and pathological stressors. In multiple animal models, LBP significantly enhances endogenous antioxidant defenses and reduces oxidative stress, effectively alleviating testicular damage induced by chemotherapeutic agents (e.g., cyclophosphamide and doxorubicin) (Qian and Yu, 2016; Xin et al., 2012), environmental toxins (e.g., bisphenol A and cadmium) (Varoni et al., 2017; Zhang et al., 2013), ionizing radiation (e.g., 60Co-γ irradiation) (Luo et al., 2011), and metabolic disturbances such as diabetes and obesity (Shi et al., 2017a; Yang et al., 2020).

LBP at doses of 0.2, 0.4, and 0.6 g/kg significantly enhanced testicular SOD activity and reduced nitric oxide (NO) levels in a dose-dependent manner, effectively mitigating cyclophosphamide-induced sperm quality deterioration. However, the underlying mechanisms regulating antioxidant enzyme expression by LBP remain to be elucidated LBP (200 mg/kg) effectively reversed doxorubicin-induced elevation of MDA levels and reduction of glutathione peroxidase (GSH-Px) activity, while increasing plasma T levels. This treatment significantly ameliorated doxorubicin-induced testicular weight loss, decreased sperm concentration and motility, and reduced abnormal sperm rates, ultimately attenuating doxorubicin-induced degenerative changes in seminiferous tubules. But the study lacked dose-gradient experiments, and the precise mechanisms underlying its antioxidant effects remain unclear (Xin et al., 2012). LBP at doses of 50, 100, and 200 mg/kg all significantly increased SOD and glutathione GSH-Px activities while reducing MDA content, effectively alleviating bisphenol A (BPA)-induced spermatogenic damage in mouse testes and significantly increasing testicular and epididymal weights. However, these protective effects did not exhibit dose-dependent enhancement. (Zhang et al., 2013). However, Qian & Yu’s study demonstrated that LBP at doses of 0.2, 0.4, and 0.6 g/kg could dose-dependently and significantly enhance testicular SOD activity while reducing nitric oxide (NO) levels, effectively mitigating cyclophosphamide-induced sperm quality deterioration (Qian and Yu, 2016). In the context of radiation-induced injury, LBP significantly antagonizes the deleterious effects of 60Co-γ irradiation on male reproductive function by enhancing antioxidant enzyme activity, reducing DNA damage, maintaining hormonal balance, and improving sexual performance (Luo et al., 2011). Additionally, LBP exhibits therapeutic efficacy in models of metabolic and aging-related testicular injury. In STZ -induced diabetic mice, LBP improves spermatogenesis and preserves testicular architecture by attenuating oxidative stress and inhibiting apoptosis (Shi et al., 2017a). In obesity-induced male infertility, LBP reduces testicular oxidative and endoplasmic reticulum (ER) stress, enhances insulin sensitivity, and restores androgen levels (Yang et al., 2020).

**FIGURE 5:**
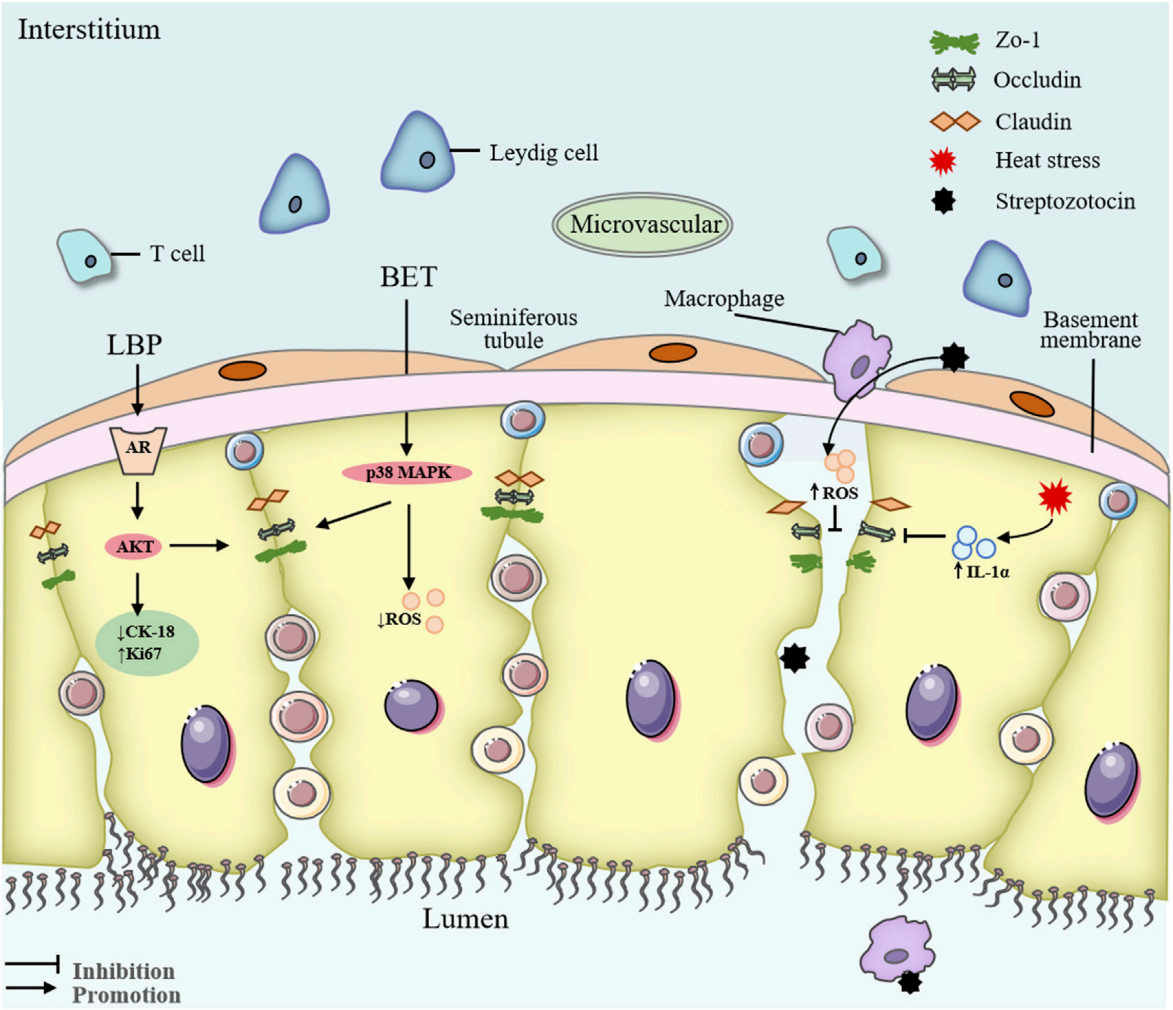
Mechanism of Lycii Fructus in repairing the blood-testis barrier. Abbreviations: LBP, *Lycium barbarum* polysaccharides; BET, Betaine; CK-18, cytokeratin-18; Ki67, proliferation marker protein Ki-67; ROS, reactive oxygen species; IL-1α, interleukin-1 alpha; AKT, protein kinase B; AR, androgen receptor.

Other metabolites of Lycii Fructus, such as *Lycium barbarum* seed oil (LBSO) and BET, also display significant antioxidative properties. LBSO alleviates oxidative stress in D-galactose-induced subacute testicular aging and TM4 Sertoli cells by suppressing mitochondrial oxidative stress through the SIRT3/AMPK/PGC-1α signaling pathway (Yang et al., 2021) (Figure 5). BET, a key active component of Lycii Fructus, protects the BTB in diabetic mice by suppressing oxidative stress and modulating the p38 MAPK signaling cascade (Jiang et al., 2019) (Figure 5). Furthermore, in a rat model of testicular torsion-reperfusion injury, Lycii Fructus extract enhance total antioxidant capacity and reduce lipid peroxidation, thus mitigating ischemia-reperfusion-induced testicular damage (Dursun et al., 2015). Collectively, these findings underscore the robust antioxidant capacity of LBP and other Lycii Fructus metabolites in counteracting testicular oxidative damage across a wide spectrum of injury models, highlighting their therapeutic potential in male reproductive disorders. Nevertheless, further research is warranted to elucidate the precise molecular targets and regulatory pathways underlying these protective effects.

### 4.3 Regulation of apoptosis

Apoptosis, a form of programmed cell death, plays a crucial role in maintaining testicular homeostasis and spermatogenesis (Cao et al., 2025). In the testis, apoptosis facilitates the removal of excess, damaged, or defective germ cells during spermatogenesis, ensuring an optimal balance between germ cell proliferation and maturation (Simões et al., 2013). However, under pathological stimuli—including diabetes, ionizing radiation, chemical or drug toxicity, and chronic psychological stress—the intrinsic (mitochondrial) and extrinsic (death-receptor) apoptotic pathways can become excessively activated, resulting in germ cell depletion, disruption of the seminiferous epithelium, Leydig and Sertoli cell dysfunction, and suppressed T biosynthesis (Yi et al., 2022). Excessive germ cell apoptosis and Sertoli cell injury impair the supportive microenvironment, while Leydig cell apoptosis reduces androgen levels, collectively impairing spermatogenesis and contributing to male infertility. Mitochondria play a central role in this process by releasing pro-apoptotic factors, such as cytochrome c, into the cytoplasm, triggering caspase cascades that lead to DNA fragmentation and cellular demise (Xu et al., 2020). ER-mediated stress pathways also contribute by upregulating CHOP and activating caspase-12, further sensitizing testicular cells to apoptotic signaling (Yang et al., 2020). Thus, regulating testicular apoptosis represents a promising therapeutic target for preserving reproductive function.

LBP has been reported to attenuate apoptosis and oxidative stress in STZ-induced diabetic mice, thereby improving spermatogenic capacity (Lei et al., 2020; Shi et al., 2018; Shi et al., 2017a). The administration of LBP (10 mg/kg) alleviates low-dose ionizing radiation-induced spermatogenic cell apoptosis by upregulating the expression of the anti-apoptotic gene Bcl-2, downregulating the pro-apoptotic gene Bax, and modulating the Bcl-2/Bax ratio. Additionally, it prevents mitochondrial membrane potential depolarization and reduces mitochondrial membrane permeability, thereby mitigating testicular morphological damage (Luo et al., 2014). However, this study failed to establish a dose-response relationship and lacked positive controls (e.g., amifostine) for comparative evaluation of LBP’s relative radioprotective efficacy. In an *in vitro* cryopreservation study, Yan et al. found that adding LBP to glycerol-egg-yolk-citrate cryopreservation medium increased anti-apoptotic Bcl-2 levels while decreasing pro-apoptotic Bax, cytochrome C, and caspase-3. The modified medium also reduced ROS production. These results suggest LBP protects mitochondrial structure and sperm function by suppressing ROS generation during freeze-thaw cycles, thereby preventing activation of mitochondrial apoptotic pathways (Yan et al., 2020). The study found that consecutive 14-day intraperitoneal administration of BET (200 mg/kg) alleviated testicular damage in male mice subjected to chronic restraint stress (CRS), significantly reducing apoptosis in seminiferous tubules and germ cells without affecting serum testosterone levels. This suggests that BET may exert its testicular protective effects through testosterone-independent pathways, offering a potential dietary intervention strategy for male fertility issues caused by psychological stress (Meng et al., 2022). Additionally, LBP protects Leydig MLTC-1 cells from cisplatin-induced injury by inhibiting ER stress-mediated apoptosis and promotes T production (Yang et al., 2018b) (Figure 4). Collectively, these findings demonstrate that LBP and its related metabolites can modulate both mitochondrial and ER-stress-mediated apoptotic pathways, thereby protecting testicular cells under various pathological insults.

### 4.4 Regulation of autophagy

Autophagy is a conserved cellular process that helps remove damaged organelles and misfolded proteins under stress conditions, thus preserving cell survival and homeostasis (Yovitania et al., 2022). In male reproductive disorders, dysregulated autophagy—either insufficient or excessive—has been implicated in impaired spermatogenesis, testicular degeneration, and hormonal imbalance (Chen et al., 2025). Therefore, restoring autophagic balance, rather than simply enhancing or inhibiting it, may help stabilize the testicular microenvironment and improve male fertility. In addition to its anti-apoptotic properties, LBP also exhibits robust autophagy-modulating effects under various pathological conditions.

In in vivo experiments, LBP, 40 mg/kg significantly improved testicular dysfunction in STZ-induced diabetic mice by upregulating the protein expression of phosphorylated PI3K and Akt, and downregulating the protein and mRNA expression of autophagy markers Beclin-1 and LC3I. These results suggest that excessive or maladaptive autophagy may contribute to testicular damage in diabetic conditions, and LBP exerts a protective effect by rebalancing autophagy. However, the study did not establish a causal link between PI3K/Akt activation and autophagy modulation (Shi et al., 2018). Similarly, di(2-ethylhexyl) phthalate (DEHP) promotes the expression of pro-apoptotic proteins such as caspase-3, caspase-8, and Bax, and exacerbates apoptosis by reducing the Bcl-2/Bax ratio, demonstrating dose-dependent toxicity. *Lycium barbarum* glycopeptide (LBGP), a glyco-conjugate further purified from LBP, is considered one of the most bioactive metabolites of Lycii Fructus (Dai et al., 2023). Zhou et al. found that 100 mg/kg LbGp alleviates testicular injury in mice by reversing DEHP-induced excessive autophagy (downregulation of SIRT1 and upregulation of FoxO3a and LC3), while showing no significant effect on cleaved caspase-3. This suggests that its protective mechanism primarily involves modulating autophagy rather than directly inhibiting apoptosis. Despite these promising results, both studies lack *in vitro* validation and genetic approaches (e.g., gene knockouts or siRNA), making it difficult to confirm the precise molecular targets involved. Future research should focus on cellular-level investigations to elucidate its molecular mechanisms (Zhou et al., 2022).

### 4.5 Regulation of inflammation

Chronic inflammation plays a pivotal role in the pathogenesis of various male reproductive disorders, particularly in pathological conditions such as testicular injury, varicocele, diabetes, and chronic stress-induced reproductive decline. Inflammatory mediators can disrupt the integrity of the BTB (Chojnacka et al., 2016), impair spermatogenesis, exacerbate oxidative stress responses, and promote apoptosis of testicular germ and supporting cells (Figure 5). Macrophages, as key immune cells resident in the testicular microenvironment, have emerged as central regulators of inflammation in male reproductive tissues (Schuppe et al., 2008). Under homeostatic conditions, testicular macrophages contribute to immune privilege and tolerance to autoantigens produced during spermatogenesis (Mosser et al., 2021). However, in response to injury or stress, macrophages become activated and secrete a range of pro-inflammatory mediators, including tumor necrosis factor-α (TNF-α), interleukin-1β (IL-1β), and interleukin-6 (IL-6), which can impair Sertoli and Leydig cell function, damage seminiferous tubules, and inhibit T synthesis (Shi et al., 2022). Experimental models of male reproductive injury have demonstrated that an imbalance in macrophage polarization—specifically an increase in pro-inflammatory M1 macrophages—is associated with more severe testicular dysfunction and reduced fertility (Balgetir et al., 2024; Li et al., 2023).

Natural metabolites with anti-inflammatory and immunomodulatory properties have garnered increasing attention in the prevention and treatment of male infertility. Studies have shown that BET can significantly improve testicular histoarchitecture and sperm quality in a CRS mouse model, restoring the structural integrity of seminiferous tubules, likely by reducing testicular oxidative stress and levels of pro-inflammatory cytokines (including TNF-α and IL-6) (Meng et al., 2022). In addition, LBP demonstrates potent immunomodulatory activity in a cyclophosphamide-induced male reproductive toxicity model. Research indicates that LBP effectively improves sperm motility, viability, and morphology, while also regulating both systemic and local cytokine levels. It promotes the expression of anti-inflammatory cytokines and inhibits the release of pro-inflammatory mediators (Qian, 2019). These findings suggest that Lycii Fructus–derived metabolites, such as BET and LBP, may help alleviate male reproductive dysfunction by modulating inflammatory responses within the testes. However, current evidence on their specific anti-inflammatory mechanisms remains limited, and further studies are needed to elucidate their immunomodulatory targets, signaling pathways, and therapeutic relevance in inflammation-driven infertility.

### 4.6 Antifibrotic effect

Testicular fibrosis is a hallmark feature of chronic testicular injury and a major contributor to irreversible spermatogenic failure. It is characterized by excessive extracellular matrix (ECM) deposition, disruption of seminiferous tubule architecture, and loss of the germ cell microenvironment (Willems et al., 2022). Persistent oxidative stress, inflammation, and apoptosis are known to promote the activation of fibrotic signaling pathways, leading to collagen accumulation and testicular degeneration. Central to this process is the activation of profibrotic cytokines, such as transforming growth factor-beta (TGF-β), as well as the activation of fibroblasts and peritubular myoid cells, which secrete abnormal amounts of collagen types I, III, and IV, along with fibronectin (Xiao et al., 2022). Dysregulated interactions between immune cells (e.g., macrophages, mast cells) and testicular somatic cells further exacerbate ECM accumulation and tissue remodeling, creating a vicious cycle that sustains fibrosis and impairs spermatogenesis (Xu et al., 2024).

Studies have demonstrated that LBP exerts protective effects against ethanol-induced testicular fibrosis and spermatogenic damage. In a chronic low-dose ethanol exposure model in mice, LBP administration significantly improved sperm quality, reduced germ cell apoptosis, and downregulated the expression of fibrosis-related genes such as Col1a1 and Col1a2 (Liu et al., 2020). Similarly, DEHP has been shown to induce abnormal collagen fiber deposition in the testicular interstitium, leading to fibrosis and impaired spermatogenesis. In animal studies, Masson staining revealed marked collagen accumulation following DEHP exposure, while intervention with LbGp significantly alleviated this deposition. Specifically, compared to the control group, the collagen volume fraction (CVF) in the DEHP group increased by 1.90% (*P* > 0.05), whereas the DEHP + LbGp group showed a 1.86% reduction in CVF compared to the DEHP group (*P* > 0.05), suggesting a trend toward antifibrotic activity of LbGp, though the changes did not reach statistical significance (Zhou et al., 2022). Moreover, Lycii Fructus appears to exert its antifibrotic effects through multiple mechanisms. First, LBP reduces oxidative stress by upregulating antioxidant enzymes, thereby mitigating ROS-mediated fibroblast activation. Second, LBP suppresses proinflammatory cytokines such as TNF-α and IL-6, both of which are known to promote a fibrotic microenvironment. Third, there is evidence that LbGp may downregulate the TGF-β signaling pathway—a canonical pathway implicated in fibrosis across multiple organs—though this mechanism requires further validation in testicular tissue (Liang et al., 2025).

## 5 Clinical study on the treatment of male infertility by Lycii Fructus

Lycii Fructus is one of the most widely used botanical drugs in TCM for the treatment of male infertility. While clinical trials focusing solely on Lycii Fructus or its extracts are limited, numerous studies have demonstrated that TCM compound formulas containing Lycii Fructus are effective and safe in the treatment of oligoasthenozoospermia. Therefore, this section reviews six clinical studies involving Lycii Fructus-containing formulas, with a focus on evaluating their clinical efficacy and safety in the treatment of male infertility (Table 3).

**TABLE 3:** Clinical trials of the effects of Lycii Fructus on male infertility.

Therapeutic drugs	Characteristic	Case	Intervention	Treatment courses	Outcomes	Safety	Ref.
Qilin Pills (QLP) (Composition: *Fructus Lycii*, *Radix Rehmanniae Praeparata*, *Fructus Corni*, *Rhizoma Dioscoreae*, *Cortex Eucommiae*, *Radix Morindae Officinalis*, *Herba Cistanches*, *Fructus Schisandrae*, *Fructus Foeniculi*, *Fructus Broussonetiae*, *Radix Achyranthis Bidentatae*, *Poria cocos*, *etc.*)	Oligoasthenospermia	168 (82 QLP/86 control)	• QLP group: 6g QLP tid • Control group: Placebo pills tid	6 months	QLP group vs*.* Control group • Sperm concentration: 25.13 vs*.* 11.62 × 10^6^/mL, (*P* < 0.01) • Sperm motility: 56.33% vs*.* 26.23%, (*P* < 0.01) • Grade a+b sperm: 33.81% vs*.* 17.32%, (*P* < 0.01) • Grade a sperm: 22.84% vs*.* 13.56%, (*P* < 0.01) • Pregnancy rate: 32.91% vs*.* 15.85%, (*P* < 0.01)	Not reported	Huang et al. (2019)
	Oligoasthenospermia	216 (108 QLP/108 control)	• QLP group: 6g QLP tid • Control group: 6g Wuzi Yanzong Pills bid	12 weeks	QLP group vs. Control group • Sperm motility: 32.95% vs*.* 25.75%, (*P* < 0.05) • Total sperm count: 205.44 vs*.* 170.18 × 10^6^/mL, (*P* < 0.05) • Progressively motile sperm: 61.10 vs*.* 50.73 × 10^6^/mL, (*P* < 0.05)	• QLP group: 4 cases of mild ALT elevation and 2 cases of mild T-Bil elevation • Control group: 2 cases of mild ALT elevation, 1 case of abnormal γ-glutamyl transferase, and 1 case of upper respiratory infection	Mao et al. (2017)
	Oligoasthenospermia	310 (208 QLP/102 control)	• QLP group: 6g QLP tid • Control group: 6g Wuzi Yanzong Pills bid	12 weeks	• Superior to control group in all semen parameters, (*P* < 0.01) • Significant improvement in all semen parameters compared to baseline, (*P* < 0.01)	• QLP group: No adverse reactions • Control group: 5 cases of mild stomach pain, 2 cases of acid reflux, and 1 case of diarrhea	Shang et al. (2011)
Yougui Capsules (Composition: *Fructus Lycii*, *Radix Aconiti Lateralis Praeparata*, *Rhizoma Dioscoreae*, *Cortex Cinnamomi*, *Fructus Corni*, *Semen Cuscutae*, *Cortex Eucommiae*, *Radix Rehmanniae Praeparata*, *Colla Cornus Cervi*, *Radix Angelicae Sinensis*)	Oligoasthenospermia	80 (40 Yougui/40 control)	• Yougui group: 1.68g Yougui Capsules tid • Control group: 6g Wuzi Yanzong Pills bid	12 weeks	Yougui group vs*.* Control group: • Sperm viability: 65.7% ± 13.1% vs*.* 38.1% ± 11.1%, (*P* < 0.05) • Grade a sperm: 22.5% ± 9.1% vs*.* 13.2% ± 6.8%, (*P* < 0.05) • Grade a+b sperm: 47.6% ± 15.8% vs*.* 24.1% ± 10.9%, (*P* < 0.05) Both groups showed significant improvement vs*.* baseline, (*P* < 0.05)	There were no obvious adverse reactions in both groups	He et al. (2012)
Qixiong Zhongzi Decoction (QZD) (Composition: *Fructus Lycii*, *Astragalus monholicus*, *Herba epimedii*, *Rehmannia glutinosa*, *Rhizoma Dioscoreae*, *Fructus corni*, *Ligusticum wallichii*, *Semen cuscutae*, *Caulis spatholobi*, *Radix achyranthisbidentatae*, *Fructus amomi*, *goldthread*)	Idiopathic asthenozoospermia	66 (33 QZD/33 control)	• QZD group: 150 mL QZD twice daily • Control group: 1 g levocarnitine oral liquid twice daily	12 weeks	QZD group vs*.* Control group • Progressive grade sperms: 22.7% ± 9.0% vs*.* 14.1% ± 8.8%, (*P* < 0.05) • Non-progressive grade sperms: 38.7% ± 14.1% vs*.* 26.2% ± 15.4%, (*P* < 0.05) • Semen volume/density: No statistical difference • Pregnancy rate: No statistical difference	• QZD group: 1 of cold and 1 of nausea • Control group: 1 of headache No obvious abnormality in their blood routine, urine routine, liver and kidney function tests in the two groups	Wang et al. (2020)
Huanshao Capsules (HSC) (Composition: *Fructus Lycii*, *Radix Polygoni Multiflori Praeparata*, *Herba Ecliptae*, *Herba Epimedii*, *Semen Cuscutae*, *Radix Cynomorii*, *Radix Codonopsis*, *Radix Curcumae*, *Fructus Rubi*, *Rhizoma Dioscoreae*, *Radix Salviae Miltiorrhizae*, *Radix Astragali*, *Radix Paeoniae Alba*, *Pericarpium Citri Reticulatae Viride*, *Fructus Mori*)	Oligoasthenospermia	190 (96 HSC/94 Control)	• HSC group: 3 capsules tid • Control group: 6g Wuzi Yanzong Pills bid	12 weeks	HSC group vs*.* Control group • Sperm concentration: 28.78 vs*.* 14.78 ×10^6^/mL, (*P* < 0.05) • Grade a sperm: 26.97% vs*.* 12.17%, (*P* < 0.05) • Progressively Motile Sperm: 47.67% vs*.* 24.78%, (*P* < 0.05) • sperm viability: 60.45% vs. 38.64%, (*P* < 0.05) HSC group vs*.* Control group • Pregnancy rate: 29.17% vs*.* 18.09%, (*P* < 0.05)	• HSC group: 3 cases of mild and transient dry mouth/throat • Control group: 4 cases of mild nausea	Yang et al. (2018a)

Abbreviation: QLP, Qilin Pills; HSC, Huanshao Capsules; QZD, Qixiong Zhongzi Decoction; ALT, alanine aminotransferase; T-Bil, Total Bilirubin; γ-GT, γ-glutamyl transferase; PMS, progressively motile sperm; bid, bis in die (twice daily); tid, ter in die (three times daily); vs*.*, versus.

### 5.1 Wuzi Yanzong Pill

Wuzi Yanzong Pill is a classical TCM compound formulas for male infertility, composed of five botanical drugs: Lycii Fructus, Cuscuta chinensis, Rubus chingii, Schisandra chinensis, and Plantago asiatica. It is widely used in the treatment of conditions such as oligospermia, asthenospermia, and reduced sexual function. In recent clinical trials of TCM treatments for male infertility, Wuzi Yanzong Pill is frequently used as a control drug, and its safety and efficacy have been well recognized. Basic research has shown that Wuzi Yanzong Pill may exert its effects by regulating testicular oxidative stress, improving mitochondrial function in sperm, and promoting the secretion of reproductive hormones (Hu et al., 2021b; Zeng et al., 2010). A meta-analysis of 11 randomized controlled trials (n = 951) demonstrated that WuZi YanZong formula as an adjuvant therapy significantly improved pregnancy rates (RR 1.68, 95% CI 1.34–2.11) and multiple semen parameters including sperm concentration (+6.87 × 10^6^/mL), total motility (+15.55%), and morphology (−10.38% abnormalities) in men with infertility (Cui et al., 2025). Wuzi Yanzong Pill is generally considered safe and well-tolerated. Most clinical trials either reported no adverse effects or failed to mention them. When reported, side effects were mild and included gastrointestinal discomfort, dizziness, and occasional allergic reactions like skin rash and itching (Cui et al., 2025). These symptoms typically resolved after discontinuing the medication. As shown in Table 3, a randomized controlled trial (n = 80) showed that after 12 weeks of treatment, Yougui Capsules (1.68 g three times daily) demonstrated superior efficacy to Wuzi Yanzong Pills (6 g twice daily) in improving sperm viability (65.7% ± 13.1% vs*.* 38.1% ± 11.1%) and progressive motility (grade a+b: 47.6% ± 15.8% vs*.* 24.1% ± 10.9%) in patients with oligoasthenospermia (all *P* < 0.05), though both treatments showed significant improvements from baseline (He et al., 2012).

### 5.2 Qilin Pill

Qilin Pill (QLP) is a well-known TCM compound formulas specifically developed for the treatment of male infertility, particularly oligoasthenospermia. Composed of multiple botanical drugs, it has been widely used in clinical practice to improve semen quality and enhance reproductive outcomes (Table 3). Several clinical trials have demonstrated its efficacy and safety in improving sperm parameters and pregnancy rates. A prospective randomized trial (n = 168) demonstrated that 6-month treatment with QLP (6 g tid) significantly improved semen parameters in oligoasthenospermic men compared to placebo, with marked increases in sperm concentration (25.13 vs. 11.62 × 10^6^/mL), total motility (56.33% vs*.* 26.23%), and pregnancy rates (32.91% vs*.* 15.85%). However, the study did not report any safety data regarding the medication (Huang et al., 2019). A multicenter randomized double-blind trial (n = 216) demonstrated that 12-week treatment with QLP (6 g tid) significantly improved semen parameters in oligoasthenospermic men compared to Wuzi Yanzong Pills controls, showing time-dependent increases in sperm motility (baseline 21.75%–32.95%), total sperm count (156.27–205.44 × 10^6^/mL), and progressively motile sperm (32.08–61.10 × 10^6^/mL). Safety analysis revealed 10 adverse events in total, with 6 cases (5.56%) in the QLP group (4 cases of mild ALT elevation and 2 cases of mild T-Bil elevation, all leading to treatment discontinuation) and 4 cases (3.74%) in the control group (2 cases of mild ALT elevation, 1 case of abnormal γ-glutamyl transferase requiring treatment cessation, and 1 case of upper respiratory infection that continued medication). The incidence of adverse events showed no statistically significant difference between groups (Mao et al., 2017). A multicenter open-label trial (n = 310 completers) demonstrated that 12-week treatment with QLP (6 g tid) significantly improved semen parameters in oligoasthenospermia patients compared to Wuzi Yanzong Pills controls (6 g bid), with superior increases in sperm concentration, grade a+b motility, and progressive motility (grade a) at all timepoints. While both groups showed improvement from baseline, the QLP group exhibited significantly greater enhancement in all semen parameters. Safety monitoring showed no adverse reactions in the QLP group, while the control group reported 5 cases of mild stomach pain, 2 cases of acid reflux, and 1 case of diarrhea - all of which were mild and did not affect treatment continuation (Shang et al., 2011).

### 5.3 Other traditional Chinese medicine (TCM) compound formulas

A randomized controlled trial (n = 66) demonstrated that compared to levocarnitine, 12-week treatment with Qixiong Zhongzi Decoction (QZD; 150 mL bid) significantly improved sperm progressive motility (22.7% ± 9.0% vs*.* 14.1% ± 8.8%) and non-progressive motility (38.7% ± 14.1% vs*.* 26.2% ± 15.4%) in patients with idiopathic asthenozoospermia (all *P* < 0.05), while no intergroup differences were observed in semen volume, density, or pregnancy rates. During treatment, two mild adverse events occurred in the QZD group (1 case of cold and 1 case of nausea), and 1 case of headache was reported in the control group, all of which resolved spontaneously without intervention (Wang et al., 2020). A multicenter randomized open-label trial (n = 190 completers) demonstrated that 12-week treatment with Huanshao Capsules (HSC; 3 capsules tid) significantly improved semen parameters in oligoasthenospermia patients with spleen-kidney asthenia compared to Wuzi Yanzong Pills (6 g bid), with time-dependent increases in sperm concentration (baseline 14.78 to 28.78 × 10^6^/mL), progressive motility [grade a: 12.17%–26.97%; Progressively Motile Sperm (PMS): 24.78%–47.67%], and viability (38.64%–60.45%). HSC also achieved higher pregnancy rates (29.17% vs. 18.09%, *P* < 0.05). Safety monitoring revealed 3 cases of mild and transient dry mouth/throat in the HSC group, which resolved with increased water intake, while the control group reported 4 cases of gastrointestinal discomfort (nausea with/without vomiting) that improved after adjusting medication timing to 30 min post-meal (Yang et al., 2018a).

Overall, although clinical trials exclusively evaluating Lycii Fructus are scarce, current evidence from compound formulas containing Lycii Fructu*s* suggests consistent improvements in semen quality and pregnancy outcomes, with a favorable safety profile. Further rigorously designed trials focusing on Lycii Fructus as a standalone intervention are needed to clarify its specific clinical efficacy.

## 6 Discussion

Lycii Fructus, as a botanical drug and a plant with both medicinal and nutritional value, was classified as a “superior-grade” medicine in the *Shennong’s Classic of Materia Medica* (Gao et al., 2017). It is renowned for its functions of “tonifying the kidney and replenishing essence” and has been used in many Asian countries to treat deficiency-related diseases (Fu et al., 2025). Modern pharmacological research has revealed that Lycii Fructus and its active metabolites, including LBP, BET, carotenoids and flavonoids, possess multiple biological properties such as antioxidant, immunomodulatory, anti-fatigue, anti-aging and reproductive protective effects, with these metabolites working synergistically to exert comprehensive health benefits (Gao et al., 2017).

In recent years, a growing body of research has focused on the protective effects of Lycii Fructus on the male reproductive system, suggesting its promising potential in the treatment of male infertility (Yang et al., 2024). This review summarizes recent *in vivo* and *in vitro* studies on the effects of goji berries and their active metabolites on male reproductive function. These studies reveal that LBP participates in spermatogenesis, testicular structural protection, and endocrine regulation through multiple pathways (Table 2), thereby improving sperm quality and alleviating reproductive dysfunctions caused by various etiologies. In terms of reproductive endocrinology, LBP has been shown to upregulate hormones associated with the HPG axis, such as GnRH, FSH, and LH, thereby promoting the synthesis and secretion of T, which is essential for spermatogenesis (Shi et al., 2017b). Regarding oxidative stress, LBP demonstrates strong free radical scavenging abilities, significantly enhancing the activities of antioxidant enzymes such as SOD, catalase (CAT), and GSH-Px in testicular tissue while reducing MDA levels. This helps to mitigate damage to spermatogonia, Sertoli cells, and Leydig cells (Meng et al., 2022). Additionally, LBP exhibits pronounced anti-apoptotic effects by upregulating the expression of Bcl-2 and downregulating Bax and Caspase-3, thus preventing excessive apoptosis of germ cells and maintaining testicular homeostasis (Hu et al., 2024). LBP and its metabolites have also been found to improve reproductive dysfunction in animal models by regulating autophagy, modulating immune responses, and inhibiting fibrosis.

These studies consistently demonstrate that Lycii Fructus promotes spermatogenesis and sperm maturation, increases intratesticular T, enhances sperm quality, and reduces oxidative damage and apoptosis in testicular tissues. It also mitigates the adverse effects of various damaging factors on the reproductive system, including drug toxicity, heat stress, radiation, and metabolic disorders. For example, across different models, LBP has been observed to enhance the activities of antioxidant enzymes such as SOD and GSH-Px, decrease MDA and ROS, and activate signaling pathways including PI3K/Akt, SIRT1/PGC-1α, and HO-1/Nrf2. These effects contribute to alleviating testicular tissue injury, improving sperm motility and morphology, and reducing apoptosis and DNA damage, thereby providing a biological basis for its reproductive protective effects.

However, current mechanistic research also has several notable limitations. First, substantial variation in extraction methods and dosing standards across different studies, and the lack of uniform guidelines, impair the comparability of results and limit their translational value. Second, most research has focused primarily on testicular protection and sperm parameter improvements, with relatively few studies evaluating sexual behavior, genetic toxicity, and offspring safety—areas that urgently require systematic investigation. Moreover, existing research has mostly centered on local testicular tissue and single pathways, lacking an integrated exploration of the hypothalamic–pituitary–gonadal axis and other systemic effects, as well as impacts on sexual behavior and embryonic genetic safety. This restricts a more complete understanding of the mechanisms by which Lycii Fructus improves male reproductive disorders. Future research should focus on developing standardized Lycii Fructus extracts and dosing protocols, systematically assessing the efficacy and safety of different doses, and identifying molecular targets and pharmacokinetic properties. In parallel, applying new omics techniques—such as transcriptomics, metabolomics, and spatial omics—will help elucidate the key molecular networks and pathways involved in mitigating male reproductive injury, while allowing a more comprehensive evaluation of sexual function, genetic stability, and long-term safety. It is also necessary to explore combination therapies with other agents, as well as novel delivery systems such as sustained-release nanoparticles, to enhance tissue specificity and bioavailability, thereby laying a solid technical foundation for translating Lycii Fructus into a standardized, clinically applicable therapeutic agent.

From a research-model perspective, LBP have shown consistent reproductive protective effects across a range of animal models, including those induced by heat stress, heavy metals, diabetes, radiation, drug toxicity, and immune injury, indicating broad adaptability and a common mechanism of action (Table 2). However, the existing studies still face notable limitations. Most of the evidence comes from animal experiments and *in vitro* research. While these models can reflect testicular damage under specific stressors to some extent, it remains unclear whether they adequately capture the complexity of the human reproductive physiological environment. Hence, relying solely on these models could introduce bias when translating findings to clinical practice, and cannot fully predict the real-world pharmacological effects of LBP on male reproductive function.

In addition, as a naturally derived polysaccharide mixture, LBP exhibits heterogeneity in molecular weight and complex structural characteristics (Zai et al., 2023), and variations in extraction and processing methods can significantly alter its composition. Current studies suggest that the multidimensional protective effects of LBP may stem from synergistic interactions among its multiple bioactive metabolites (including polysaccharides, flavonoids, and trace elements). We propose the following mechanistic hypothesis: these active metabolites may collectively exert beneficial effects through complementary mechanisms—the polysaccharide fraction enhances antioxidant defense systems and modulates immune responses, flavonoids further scavenge reactive oxygen species and suppress inflammatory reactions, while trace elements provide essential support for energy metabolism and spermatogenesis. These synergistic effects likely converge on key signaling pathways (e.g., the PI3K/Akt signaling cascade), effectively maintaining testicular redox homeostasis, inhibiting germ cell apoptosis, and promoting germ cell proliferation and maturation. Elucidating these synergistic mechanisms is not only crucial for comprehensively understanding LBP’s fundamental actions but will also provide important theoretical foundations for precision research and clinical translation in male reproductive health.

Current clinical data consistently demonstrate that TCM formulations containing Lycii Fructus (such as Wuzi Yanzong Pills and QLP) can significantly improve semen parameters (including sperm concentration and motility) in patients with oligoasthenozoospermia, while exhibiting generally favorable short-term safety profiles characterized primarily by mild gastrointestinal discomfort and occasional transient liver function abnormalities. However, these studies fail to elucidate the specific contribution of Lycii Fructus as a single agent within compound formulations, leaving its independent therapeutic efficacy and dose-response relationship unclear. Furthermore, evidence regarding long-term safety remains lacking, particularly concerning potential risks to hepatic function and reproductive genetic toxicity. Although the multi-target regulatory advantages of compound formulations are well-established (e.g., antioxidant effects, hormonal regulation, and mitochondrial function improvement), observed cases of elevated liver enzymes in QLP studies warrant caution regarding hepatotoxicity risks. In clinical practice, male infertility patients often require prolonged medication cycles, with some cases necessitating combination therapies - underscoring the need for robust evidence to guide rational clinical decision-making regarding Lycii Fructus use. Future research should prioritize mechanistic studies of active metabolites in Lycii Fructus and conduct long-term follow-up assessments of live birth rates and offspring health outcomes.

In conclusion, Lycii Fructus possesses multiple mechanisms of action including regulation of reproductive hormones, antioxidation, and anti-apoptosis, effectively improving male reproductive function under various pathological conditions. These findings provide strong evidence supporting its translational potential as a natural therapeutic agent for male infertility. Future efforts should prioritize rigorous clinical trials and standardized extract development, paving the way for Lycii Fructus to evolve into a safe, evidence-based phytotherapeutic agent or functional food for managing male infertility.
