# Correction: Therapeutic mechanisms of Lycii Fructus in male infertility: a comprehensive review

**DOI:** 10.3389/fphar.2025.1711381

**Published:** 2025-10-07

**Authors:** Dongyue Ma, Dexiu Li, Guanchao Du, Shengjing Liu, Anmin Wang, Hongyuan Chang, Hui Lv, Hao Wang, Fu Wang, Jun Guo

**Affiliations:** ^1^ Xiyuan Hospital of Clinical Medical College of Beijing University of Chinese Medicine, Beijing, China; ^2^ Department of Andrology, Xiyuan Hospital, China Academy of Chinese Medical Sciences, Beijing, China; ^3^ Department of Cardiology, Xiyuan Hospital, China Academy of Chinese Medical Sciences, Beijing, China

**Keywords:** Lycii Fructus, Lycium barbarum, male infertility, testis, sperm quality, oxidative stress, reproductive protection

**Affiliation 1** and **2** were swapped. Furthermore, **Affiliation 1** was erroneously given as “Department of Graduate School, Institute of Chinese Materia Medica, Beijing University of Chinese Medicine, Beijing, China”. This has been updated to “Xiyuan Hospital of Clinical Medical College of Beijing University of Chinese Medicine, Beijing, China” for author **Dongyue Ma**.

The original article has been updated.

